# Fabrication of Beeswax–Soapwort Root Powder–Gelatin Bigel-Based Foamed Emulsions for Use as a Fat Replacer in Mousse

**DOI:** 10.3390/gels12090810

**Published:** 2026-09-03

**Authors:** Alican Akcicek

**Affiliations:** Department of Gastronomy and Culinary Arts, Faculty of Tourism, Kocaeli University, Kartepe, 41080 Kocaeli, Turkey; a.akcicek@kocaeli.edu.tr; Tel.: +90-545-849-67-75

**Keywords:** bigel, gelatin, soapwort root powder, emulsion, mousse, fat replacer

## Abstract

In this study, beeswax (BW) and gelatin, soapwort root powder (SRP) were employed to create oleogel and hydrogel for bigel development, respectively. The study aimed to determine the potential utilization of SRP in the bigel system to create a novel fat replacer (bigel-based foamed emulsion) for mousse production. Bigels with 2% SRP showed a bicontinuous emulsion structure. The FTIR spectra of all the bigels exhibited no new peaks. Bigels had solid-like properties, given that no crossover point was present and *G*′ values were uniformly greater than *G*″ values. Hardness, gumminess, and chewiness were improved by increasing the bigel’s gelatin and SRP concentrations. A rise in the SRP ratio and gelatin content resulted in a higher overrun of bigel-based foamed emulsions. An increment in the SRP ratio resulted in enhanced thermal stability, with the exception of 9% G-2. The *G*′ values surpassed the *G*″ values, indicating that the mousse samples exhibited solid-like characteristics. From the prepared samples, 6% G-2 M was determined to be the closest to the control mousse in terms of hardness, springiness, cohesiveness, and gumminess values when comparing the bigel mousse samples with the control mousse sample (*p* > 0.05). The 6% G-2 M sample showed the lowest ΔE* value and was the closest sample to CM.

## 1. Introduction

Solid and semi-solid fats are essential for flavor, texture, and structure in a variety of food items [1]. Due to their elevated saturated fatty acid content, these solid fats are more prone to contributing to diet-related chronic illnesses, including obesity and diabetes [2,3]. Conventional solid fats are crucial to the structure and texture of food, but their high amounts of saturated and trans fats, as well as their effects on the environment, raise questions about sustainability and health [4]. Research initiatives have escalated to create better fat substitutes that mitigate nutritional hazards while preserving the sensory and technical properties of traditional solid fats [5,6].

Recently, researchers have concentrated on discovering fat substitutes that provide structured lipids with reduced caloric content and few or no saturated/trans fatty acids [7]. As consumers become more aware of the health dangers associated with saturated fats, demand for alternatives grows. Nonetheless, substituting solid fats with liquid oils in food compositions poses technological difficulties [1]. A possible option for generating healthier solid fats is to transform unsaturated fatty acid-rich edible oils using oleogelation, a triglyceride-free fat structuring technique [8]. Oleogels have a fat retention rate of up to 90%. However, this characteristic also causes oxidative instability and excessive lipid absorption, which eventually lowers the quality of the final product [3,9].

Bigels (BGs), two-phase gels made by mixing oleogels (OG) and hydrogels (HG), have gained interest as a solution to these problems [1]. Bigels combine the oil-binding capacity and mechanical strength of oleogels with the flexibility and water-retention capabilities of hydrogels [5]. This system exhibits far greater mechanical strength and structural stability, effectively compensating for the performance deficiencies of single hydrogels and oleogels through the formation of an interwoven network structure [10]. Bigels can imitate the mouthfeel of full-fat items by imitating fat tissue in low-fat diets [6,11]. The structure of the bigels and the oleogel-hydrogel ratio can be changed to enhance the texture of the food, for example, by thickening or adding elasticity [6]. Bigels have been used as a fat replacer in cookies [12], croissant [13], hamburger patties [14], bread [3], puff pastry [15], and low fat cream [16].

Despite bigels’ potential viscoelastic and structural qualities, research into their aeration-related applications has been restricted [17]. Furthermore, much of the present research emphasizes bigels as static oil matrices, without considering their potential to stabilize air phases under thermal circumstances and dynamic mechanical [5]. Bigel-based foamed emulsions (BFEs) have recently been recognized as a potential type of structured aerated system. In BFEs, the hydrogel phase improves foam stability during processing and baking by limiting aqueous drainage and increasing volumetric viscoelasticity, while the oleogel phase fortifies air-oil contacts [5]. BFEs have been used as fat replacers in muffins [5,17,18].

Aerated foods like mousses, creams, and ice creams are becoming more and more popular these days because of their low energy density, improved taste release, and light textures [18,19,20]. BFEs minimize the need for extra synthetic emulsifiers while offering improved aeration stability and a smooth, creamy texture because of their dual-structuring capabilities. BFEs are intriguing options for creating healthy aerated food with desired sensory qualities because of their multifunctional activity [17].

The choice of gelators former has a significant impact on the performance of bigels and BFEs [17]. Beeswax has been used as an oleogelator and gelatin has been used as a hydrogelator in bigel systems.

Beeswax is frequently utilized in oleogel formulations due to its self-emulsifying qualities, edibility, non-toxicity, cost-effectiveness, and health advantages [21,22]. Gelatin is a natural polymer that finds widespread usage in the culinary, pharmaceutical, and cosmetic sectors. Gelatin, a biodegradable substance, has good biocompatibility, is generally accepted by the human body, and decomposes easily in the environment, limiting its impact on the ecological balance [6,23].

Soapwort root powder is a *Gypsophila* sp. extract obtained from perennial herbaceous plants of the genus *Gypsophila* of the Caryophyllaceae family. The plant features a taproot and produces white flowers in June and July [24,25]. Soapwort root is distinguished from other natural resources by its elevated saponin content of 21% [26]. Saponins are glycosides derived from soapwort, characterized by surfactant characteristics and including a polycyclic aglycone known as sapogenin. A defining trait of saponins is their capacity to generate a stable, soap-like foam in aqueous solutions [27].

Mousse is an excellent food model for assessing BFEs since its quality changes greatly depending on how it is stabilized, especially during air addition and mixing. No prior research has examined at BFEs as a cream alternative in mousses, despite the rising interest in bigel technology. In this work, bigels with a 50% oleogel–hydrogel matrix were created utilizing two different gelatin (6 and 9 g/100 g) and SRP concentrations and BW as an oleogel former. After being whipped to create BFEs, these bigels were thoroughly assessed for foam stability, thermal stability, and storage stability. The BFEs were then added to whipping compositions for use as a cream substitute, and the quality of the final product—including frequency scanning, textural, and color characteristics—was evaluated. It was hypothesized that that the use of SRP–gelatin hydrogel and beeswax oleogel-structured bigel-based foamed emulsions as fat alternatives in mousse formulation would provide comparable or enhanced rheological and textural characteristics relative to the control mousse. Consequently, the study aimed to fabricate a bigel-based foamed emulsion by using beeswax oleogel gelatin hydrogel with or without soapwort root powder. Then, the BFEs were used in mousse dessert as cream fat replacer. This study scientifically introduces a polymer-based bigel-based foamed emulsion for use as a fat replacer in a food matrix. It also discusses the impact of polymers on the textural and rheological properties of BFEs and mousses. Although mousse was selected as a model food application, BFEs have potential applications as fat replacers in semi-solid and aerated foods and offer a practical solution for obtaining reduced-fat foods.

## 2. Materials and Methods

### 2.1. Materials

The bigel and mousse formulation components including avocado oil, cream (35% fat), sucrose, and nonfat milk were purchased from Turkish local food stores. Soapwort root powder (*Gypsophila* sp. extract) was acquired from Istanbul Tarım, beeswax was obtained from Petekoğlu. Gelatin (260–280 Bloom) was purchased from Gerede Jelatin (Bolu, Türkiye).All chemicals were of analytical grade.

### 2.2. Methods

#### 2.2.1. Preparation of Bigel

The bigel samples were created using modified techniques [6,28]. Gelatin powder at 6% and 9% (*w*/*w*) was dissolved in distilled water for 15 min. The hydrogel samples were created by stirring for 15 min at 90 °C and 1000 rpm using a magnetic stirrer. After adding 1% and 2% (*w*/*w*) soapwort root powder to the gelatin solution, the mixture was agitated for five minutes. The oleogel was made by dissolving 8% (*w*/*w*) beeswax in avocado oil at 90 °C and stirring at 500 rpm until the beeswax was fully melted. The oleogel and hydrogel samples were kept at +4 °C overnight. Bigel samples were then made using a 50–50% ratio of hydrogel and oleogel. The oleogel and hydrogel samples were heated, then homogenized with an digital homogenizer disperser (Daihan HG-15D, Gangwondo, Republic of Korea) at 10,000 rpm for 3 min. The bigel samples were then allowed to cool to room temperature and stored for a day at +4 °C. Prior to analysis, the samples were kept at 4 °C. The bigel sample formulations are described in Table 1.

#### 2.2.2. FTIR

The gels’ stability and compatibility were assessed using a Bruker Tensor 27 FTIR spectrometer (Bremen, Germany) equipped with an ATR attachment with a diamond crystal modulus. The detector was a DLaTGS detector fitted with a KBr beam splitter. The HG, OG, and BG samples’ FTIR spectra were obtained at a resolution of 4 cm^−1^ in the 4000–600 cm^−1^ range, with 16 scans in total for each spectrum [29]. Bruker GmbH’s OPUS application for Windows, version 7.2, was used to confirm the constitution of the gel samples.

#### 2.2.3. Rheological Properties

BG samples’ rheological characteristics were assessed with a rheometer (MCR 302; Anton Paar, Graz, Austria) configured with a temperature- and stress-controlled Peltier heating system. A PP25 rheometer probe was inserted into the parallel plate, with a 1.5 mm and 1 mm gap between the sample plates for bigel (frequency sweep) and mousse (frequency sweep), respectively. Rheological analysis was carried out at 25 °C.

Dynamic rheological analyses were conducted on BG samples using a parallel plate technique.

First, the linear viscoelastic region (LVR) was identified by an amplitude sweep test with a strain value of 0.1%. The linear viscoelastic region was determined by a strain sweep test (0.01% and 100% strain range and 1 Hz constant frequency). A strain level of 0.1% within the LVR was chosen to ensure that the samples would not deteriorate or be damaged during the subsequent frequency sweep test. The frequency sweep test was performed on LVR in the frequency range of 0.628–62.8 rad/s (0.1–10 Hz) to determine the storage modulus (*G*′) and loss modulus (*G*″) as functions of angular frequency.

Nonlinear regression and a power-law model were used to calculate dynamic rheological parameters [30].

(1) $$G^{\prime }=K^{\prime }(\omega {)}^{n^{\prime }}$$

(2) $$G^{\prime \prime }=K^{\prime \prime }(\omega {)}^{n^{\prime \prime }}$$

where *G*′ and *G*″ represent the storage and loss moduli, respectively; *ω* represents the angular frequency (s^−1^); *K*′ and *K*″ represent the consistency coefficients; and *n*′ and *n*″ represent the flow behavior indices.

#### 2.2.4. Visual Observation and Microscopy

Images of the BGs, BFEs, and mousse samples were taken using a smartphone (Redmi Note 14 Pro, Xiaomi Corp., Beijing, China). The samples were examined using a light microscope. A small quantity of bigel was put on a glass slide for microscopy and protected with a cover slip. The samples’ photos were taken at a magnification of 40× (Nikon ECLIPSE E100, Tokyo, Japan).

#### 2.2.5. Texture Profile Analysis of Bigels

A texture analyzer (TA.XT2 Plus, Godalming, UK) was used to assess the BGs’ hardness, gumminess, cohesiveness, chewiness, and springiness. The samples were tested using a cylindrical probe P/5 with a diameter of 5 mm. TPA was carried out in a two-cycle compression test, with a 5 s rest period in between cycles and distance of 10 mm, using a sample size of around 50 g in a 210 mL cylindrial glass jar.

The trigger force was 0.1 g, the pre-test speed was 2 mm/s, the test speed was 2 mm/s, and the post-test speed was 2 mm/s.

#### 2.2.6. Preparation of Bigel-Based Foamed Emulsions

Bigel-based foamed emulsions were produced by modifying a previous method [17]. Bigel samples were heated to 40 °C. Then, using a kitchen mixer (Arçelik A.Ş., K1260 RHB, Istanbul, Türkiye) fitted with a wire whisk, bigel samples were kept in an ice bath and whipped for ten minutes at maximum speed (700 watts). The resultant BFEs were examined directly following whipping.

#### 2.2.7. Characterization of Bigel-Based Foamed Emulsion

##### Foamability

The volume expansion of the BFEs was used to determine the level of aeration in the bigel foams. Samples of foam were moved into falcon tubes and weighed. The following formula was used to test foaming ability:

(3) $$Overrun\;(\%)=\left(\frac{\left(Mb\right)-\left(Mf\right)}{\left(Mf\right)}\right)\times 100$$

where Mb represents the initial starting weight of the unwhipped bigel and Mf represents the weight of the whipped foam [5,31].

##### Foam Stability (Thermal Stability and Storage Stability)

*Thermal Stability*

The BFEs’ thermal stability was assessed by modifying Tirgarian and Farmani’s (2023) methodology [17,32]. Freshly prepared foam was placed in volumetric tubes and incubated in a temperature-controlled water bath at 60 °C for ten minutes. The volume of the filtered serum (oil + water) was measured after it had cooled to room temperature. Thermal stability was calculated using Equation (4).

(4) $$Thermal\;Stability\;(\%)=\left(\frac{\left(Wt\right)-\left(Wr\right)}{\left(Wt\right)}\right)\times 100$$

where the initial weight of the BFE’s is represented by Wt and the released oil + mixture is represented by Wr.

*Storage Stability*

A 50 mL centrifuge tube containing each sample was kept for 21 days to assess the stability over time of BFEs at room temperature (which is approximately 20 °C). By measuring the amount of serum that separated at the tube’s bottom and tracking variations in the volume of foam following the storage time, storage stability was evaluated [17,31].

#### 2.2.8. Preparation of Mousse

The method described by [33] was modified and employed in the preparation of the mousses. The mousse samples were prepared using 8 g of unfatted milk, 2 g of sugar, 1 g of gelatin, and 20 g of cream or bigel-based foamed emulsion. In the preparation of the mousse, 20 g of BFE was utilized in place of 20 g of cream; given that the cream contained 35% fat, the overall fat level amounted to 7 g. Nevertheless, 20 g of BFE was used to attain the whipped cream attributes and the intricate composition of the cream. In total, 20 g of BFE comprised 10 g of oleogel and 10 g of hydrogel; 9.2 g of the 10 g of oleogel consisted of avocado oil. The calculated fat content of the cream was 7 g, whereas the determined avocado oil content of the BFE was 9.2 g. Initially, the sugar and milk were combined and dissolved by stirring at 80 °C. After that, the mixture was added to gelatin and stirred to dissolve it. The mixture was then added to the whipped bigel-based foamed emulsion (10 min) or whipped cream (for 5 min) by gentle folding using a spatula until homogenous, after it had cooled to 30 °C. Before being examined, the produced mousses were kept overnight at 4 °C. To ensure fair comparison at maximum foam overrun without structural collapse (over-whipping), the control sample (cream) was whipped for 5 min, whereas the foamed emulsions based on bigel-containing formulations were whipped for 10 min until peak aeration was achieved.

#### 2.2.9. The Rheological Properties of Mousse Samples

Dynamic rheological analyses were conducted on mousse samples using a parallel plate technique. The analysis details were the same as those given in the section describing the rheological properties of the bigels.

##### 2.2.10. The Textural Properties of the Mousse Samples

The mousse samples’ textural profile was determined by modifying the method described by Jin et al. (2026) [33]. A TA-XT Plus Texture Analyzer (Stable Microsystems, Godalming, UK) was used to conduct measurements at 20 °C. A cylindrical P/5 probe was used in this study. TPA was carried out in a two-cycle compression test, with a 5 s rest period in between cycles and a 50% strain level. The assessment used 50 mL beakers with a sample size of around 30 g. Using a trigger force of 0.049 N (5 g), the pre-test speed was 3 mm/s, the test speed was 5 mm/s and the post-test speed was 5 mm/s.

##### 2.2.11. The Color Properties of the Mousse

A colorimeter (Konica Minolta CR-400, Tokyo, Japan) was used to measure the color parameters L*, a*, and b*. Color analysis assessed the brightness (L*), redness (a*), and yellowness (b*) values following room temperature calibration. A white ceramic plate was used for calibration. The bigels and cream-made mousses’ color difference (ΔE*) was determined as follows [34]:

(5) $$∆E*\;=\;\sqrt{{\left(L_{0}^{*}-L^{*}\right)}^{2}+{\left(a_{0}^{*}-a^{*}\right)}^{2}+{\left(b_{0}^{*}-b^{*}\right)}^{2}}\;$$

where ${L_{0}}^{*}$, ${a_{0}}^{*}$, and ${b_{0}}^{*}$ are the colorimetric characteristics of cream-containing mousse samples, and L*, a*, and b* are the characteristics of mousses made with the BFEs.

##### Statistical Analysis

The mean ± standard deviation (SD) was used to present all data. All formulations were prepared, and analytical measurements were performed in triplicate (*n* = 3). The data collected at the conclusion of the study were statistically analyzed using the JMP 19 (v19) software package. Two-way analysis of variance (ANOVA) (*p* < 0.05) and the Tukey–Kramer HSD comparison test were used to assess the difference between group means. Dunnett’s post hoc test was used to statistically compare the BFE’s based mousse with the control group (CM). The parameters of the power law model were determined via nonlinear regression analysis, using STATISTICA software (v8.0) (StatSoft Inc., Tulsa, OK, USA).

## 3. Results and Discussion

### 3.1. Visual Observation and Microstructure Properties

**Figure 1:**
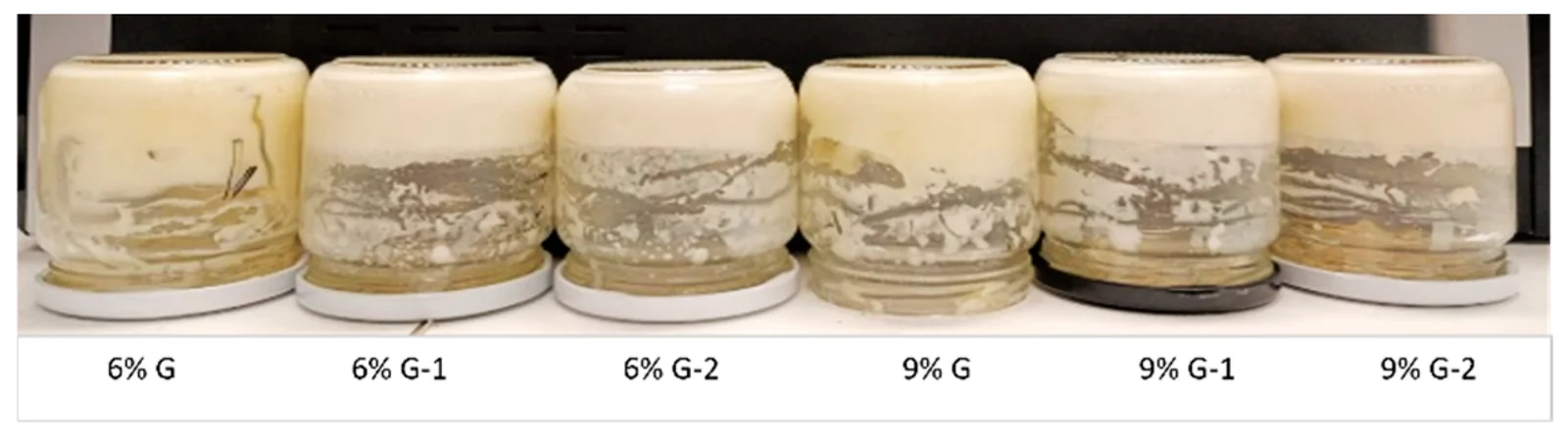
Visual Appearance of Bigel Samples.

The visual appearance of the bigels is illustrated in Figure 1. The most common method for confirming gelation and inversion stability is the tube inversion test, which involves turning a test tube and determining whether the substance flows under its own weight [35,36]. An inversion test was used to evaluate the bigels’ structural stability (phase decomposition), and the results showed that each bigel had self-standing capabilities. The samples were inverted in plastic tubes to confirm gel formation; no gravity flow was seen during this process. All samples had a yellowish color. The OG:HG ratio, the dispersed phase size distribution, and the light diffraction of the oil droplets all influence the color of bigels [37,38].

All bigel formulations had a similar appearance—opaque and yellowish—across every gelatin concentration. The bigels did not flow and maintain their form after gelation, regardless of the gelatin concentration or SRP ratio. According to [39], all gelatin concentrations produced formulations for novel rice bran wax/gelatin bigels that looked comparable, were opaque, and were white. Additionally, that previous study observed that the bigels did not show fluidity after gelling and retained their form irrespective of the gelatin concentration or OG:HG ratio.

In the present study, every sample was solid to the touch and had a smooth surface. Similar results have been recorded for bigels based on monoglyceride oleogel and k-carrageenan hydrogel and for bigels based on monoglycerides in olive oil oleogels and gelatin and κ-carrageenan hydrogels, respectively [38,40].

**Figure 2:**
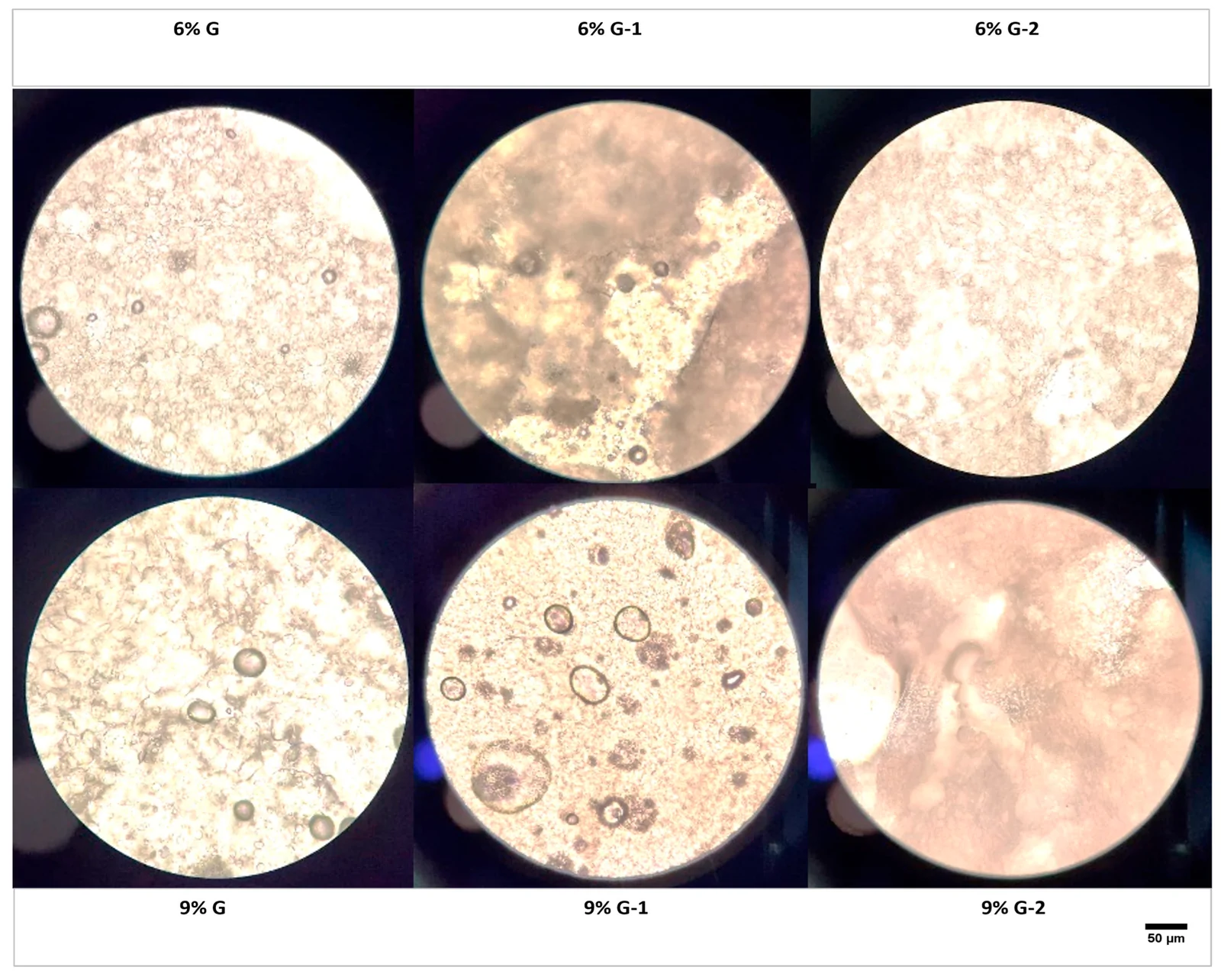
The Microstructural Characteristics of Bigels.

Figure 2 showed the microstructural properties of bigels. The microstructure of bigels were opaque, with uniformly shaped droplets that varied in size. The microstructure of 6%G, 6%G-1 and 9%G, 9%G-1 was opaque and had bigger droplets, indicating that the hydrogel was in the oleogel. The hydrogel, which had disintegrated into particles, was found to be encircled by a “liquid” oleogel whose structure had changed over time. Similar findings were recorded by Fasolin et al. (2021) [41], who reported that the microstructure of gellan gum–glycerol monostearate bigels comprised hydrogel particles scattered inside a continuous organogel matrix.

Bigels with 2% SRP showed reduced droplet size and no distinct barrier between the oleogel and hydrogel phases. Moreover, the oleogel and hydrogel phases were not clearly separated; rather, they interacted synergistically due to the uneven distribution of one over the other.

### 3.2. FTIR Spectra of Bigels

**Figure 3:**
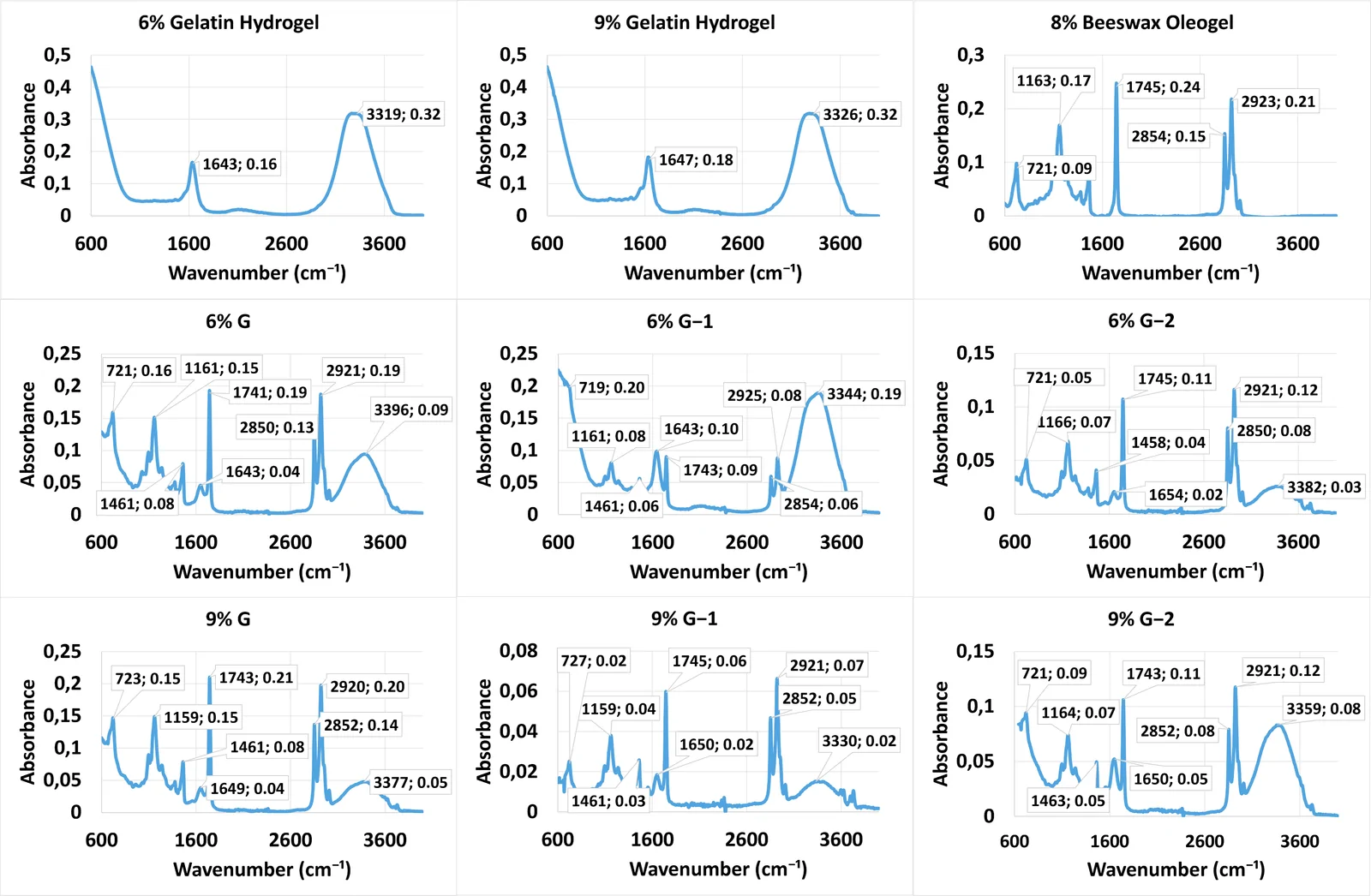
The FTIR spectrum of the Bigel Samples.

Figure 3 displays the FTIR spectra of the bigels. The C–H vibration of CH_2_ is the source of the peak at 719–727 cm^−1^ [7]. The amide I area (1700–1600 cm^−1^) showed a dense and visible band, as would be expected for a protein-based gel [38]. The amide I vibration of the peptide bonds in gelatin is responsible for the absorption peaks at 1164 cm^−1^ and 1638 cm^−1^ (C=O stretching) [6,7,39]. The existence of ester compounds in the bigels is indicated by the presence of C=O stretching vibration peaks in the area between 1737 and 1745 cm^−1^ [6,42]. The absorption peaks at 2852 cm^−1^ and 2921 cm^−1^ are indicative of stretching vibrations of C–H bonds in the samples’ hydrocarbon chains [6,43].

The hydrogel’s intramolecular hydrogen bonding caused an O-H bending vibration peak to appear between 1643 and 1654 cm^−1^ [6]. The protein’s amide I band experienced a shift, which may have been caused by the intensified electrostatic contact with the polysaccharide [44,45].

All of the bigels showed a wide band at 3600–3000 cm^−1^, which may have been caused by the O–H stretching vibrations involved in hydrogen bonding inside the hydrogel and the heads of fatty acyl molecules in the oleogel, in line with reports in the literature [44,46].

A strong, distinctive peak spanning from 3330 cm^−1^ to 3382 cm^−1^ was seen in all bigel samples. This peak was caused by O-H stretching vibrations and suggests that the bigels possessed a comparable hydrogen-bond network structure [6,47]. The broad peak at around 3370 cm^−1^ is linked to O–H stretching, N–H stretching, and hydrogen bonding [43]. Due to the high gelatin concentration of the samples, each bigel sample exhibited special, gelatin-specific peaks [39]. In accordance with [6], it was observed that the hydrogel’s distinctive peaks moved in the bigel; this may be explained by the hydrogel forming hydrogen bonds with the bigel’s lipid molecules, which in turn caused a shift in the O-H groups’ vibrational frequency.

As documented previously in the literature, the FTIR spectrum of soapwort root extract is generally similar to that of edible vegetable oils. The region at 1710 cm^−1^ is the saponin (C=O) absorbance and indicates the fundamental difference from oils [48]. Increasing the SRP concentration from 1 to 2 resulted in an enhanced absorbance intensity for the saponin molecule (1710 cm^−1^), causing it to overlap with the ester peak of the oleogel (1737 and 1745 cm^−1^). Also, an increase in SRP ratio from 1 to 2 led to an increase in absorbance intensity at 2850 cm^−1^ and 2921 cm^−1^. Except for the peaks at 1643 cm^−1^ and 3344 cm^−1^ for the 6% G-1 sample, the total absorbance values were typically reduced when 1% SRP was added to the bigels.

In the series of six bigels, SRP addition exhibited a phase-dependent pattern. Adding 1% SRP decreased absorbance in oleogel bands (1743, 2850, and 2921 cm^−1^) while increasing absorbance in hydrogel bands (1643 and 3328 cm^−1^). This trend reversed with the addition of 2% SRP; hydrogel absorbance decreased and oleogel absorbance increased. In the series of nine bigels, adding 1% SRP resulted in a general decrease in absorbance across all oleogel and hydrogel bands. Adding 2% SRP altered this profile, leading to an increase in absorbance.

These concentration-dependent shifts were attributed to intermolecular hydrogen bonds and hydrophobic interactions.

The FTIR spectra of all the bigel samples showed no new peaks [7]. The FTIR spectra showed no new characteristic peaks upon bigel formation or SRP involvement; this suggests that the components interacted primarily through weak non-covalent forces (hydrogen bonds and hydrophobic interactions) without forming covalent bonds. Analysis of the bigel samples showed the wavelengths present in the hydrogel and oleogel samples, and no new peak appeared in the spectra. Similarly to the findings reported by Habibullah et al. (2025) [49], non-covalent interactions (van der Waal forces, hydrogen bonding) perform an essential role for maintaining the structural integrity of hydrogels and oleogels in bigel formulations.

### 3.3. Rheological Properties of Bigels

**Figure 4:**
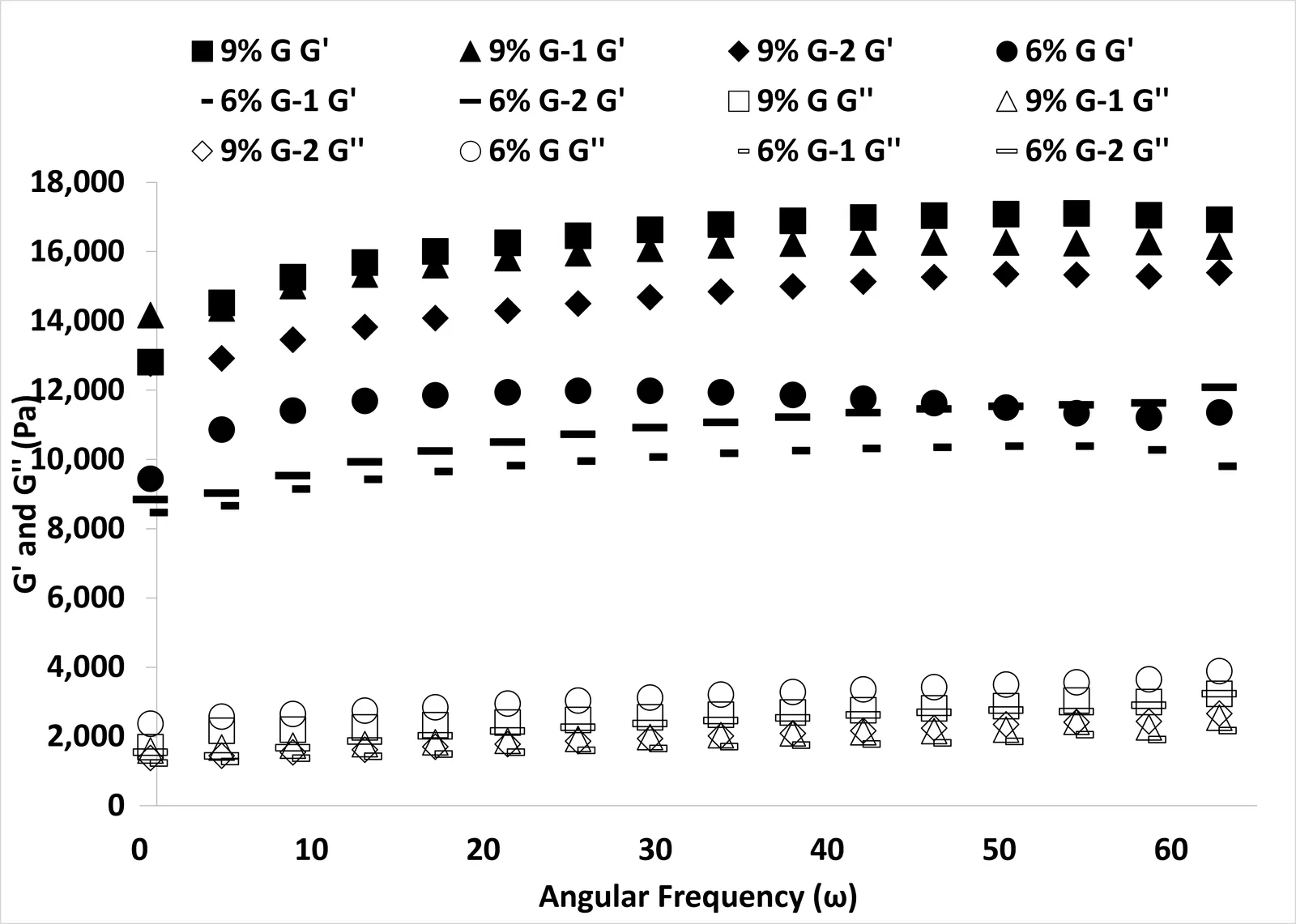
The Viscoelastic Properties of the Bigel Samples (mean, *n* = 3).

As seen in Figure 4, the bigels’ storage modulus (*G*′) was consistently higher than its loss modulus (*G*″). Bigel samples exhibited solid-like behavior, since there was no crossover point throughout the test and *G*′ values were constantly greater than *G*″ values [28].

Figure 4 shows that the bigel samples’ *G*′ changed with the frequency increase. Similar findings were documented by Zheng et al. (2020) [40] for κ-carrageenan hydrogel and monoglyceride oleogels as carriers for β-carotene.

Comparing all the bigel samples, 9% G had higher *G*′ values and 6% G-1 had lower *G*′ values. Figure 4 showed that *G*′ values decreased at gelatin concentrations of 6% and 9% when the SRP ratio in the bigel samples was increased at higher frequencies, except for the 6% G-2 samples. Consistent with the previous literature, this could have been associated with the protein’s deeper penetration into the lipid phase and the persistent connections between free surfactant molecules and protein–surfactant complexes at the interface, which stabilized the interface and prevented surfactant molecules from completely displacing the protein [50]. As previously reported in the literature, when the surfactant concentration rises, the viscoelastic modulus at the surface falls. Modifications to the characteristics of the protein–surfactant complex itself could be attributed to this rheological decrease at the water/oil interface [50], which could help explain the reduced *G*′ values.

*G*′ values were greater in the 9% G bigel samples than in the 6% G bigel samples. Bigel strength increased when the gelatin concentration was raised. This could be explained by hydrogen bonding and van der Waals forces. Gelatin, a hydrophilic protein that forms robust networks, established a dense three-dimensional structure with the crystalline frameworks of beeswax in the oleogel. This increased the system’s viscoelastic properties and made it more rigid, enabling it to reach the maximum levels of loss and storage modulus [6,51]. The Power Law model was used to compare the findings on the dynamic rheological characteristics of the bigels. This model correctly simulated the dynamic rheological characteristics of the *K*′ values of the bigel samples, except for 6% G, and the *K*″ values of the bigel samples, except for 9% G-1 and 6% G-1, as seen in Table 2. K’ values were determined to be higher than *K*″ values in every sample, confirming viscoelastic solid behavior. The samples were viscoelastic solids, as indicated by the findings for *n*′ and *n*″ values.

The *K*′ values of the bigel samples ranged from 8312.95 to 14,019.4 Pa·s. The 9% G-1 showed the highest *K*′ value compared with the other bigel samples. Even though the samples differed numerically, the main effect of SRP ratio did not significantly affect the *K*′ value. The samples’ *K*″ values were not statistically significant, with the exception of the 2% SRP concentration. The *K*′ values of the 9% G-1 samples were greater than the 6% G, 6% G-1, and 6% G-2 samples (*p* < 0.05). The influence of the gelatin–SRP concentration might explain this finding with regard to the *K*′ values of the samples (*p* < 0.05).

### 3.4. Texture Profile of Bigels

Table 3 displays the bigels’ textural characteristics, including gumminess, chewiness, springiness, cohesiveness, and hardness. The main influence of the gelatin concentration and the main effect of SRP ratio on the hardness, gumminess, and chewiness values was found to be statistically significant (*p* < 0.05). Hardness, gumminess, and chewiness were improved by increasing the bigels’ gelatin and SRP concentrations. The increased gel strength and viscoelastic network were attributed to the incorporation of SRP and gelatin potential function as active fillers, through hydrogen bonding and/or hydrophobic interactions [51]. The bigels’ discrete phase functioned as an “active filler,” enhancing hardness and enabling better gel structure [52,53]. As active fillers, hydrogel droplets trapped in the bigel matrix strengthen the network structure and increase the gel’s strength [7,54].

The hardness value rose in accordance with the bigels’ SRP concentration. In the same way, hardness values increased as the gelatine concentration in the bigel increased. Bigel with 9% G-2 was the hardest sample, while 6% G was the softest. The primary cause of this phenomenon is that the gelatin within the hydrogel established a compact cross-linked structure via intermolecular hydrogen bonds and van der Waals interactions, resulting in increased hardness of the bigel [6]. Research indicated that substituting soapwort extract for albumin in marshmallow manufacture resulted in a nearly twofold increase in hardness when soapwort extract constituted up to 50% of the mixture, compared to a 100% albumin ratio [55]. Çam and Topuz (2018) [24] reported that the highest hardness value was seen in a halva sample made with soapwort powder.

The hardness, gumminess, and chewiness values of the 9% G-2 samples were greater than those of the other bigel samples (*p* < 0.05). The influence of the gelatin–SRP concentration might explain this finding regarding the hardness, gumminess, and chewiness values of the samples (*p* < 0.05).

The cohesiveness values of the bigels were found to be between 0.476–0.569. The effect on gelatine concentration on cohesiveness values was found to be statistically significant. Increased hydrogel concentration resulted in the establishment of a continuous network, enhancing both adhesivenes and cohesiveness [46,52].

All bigel samples showed high springiness values except 9% G-1. Minimal spring values suggest inadequate shape recovery following deformation [56]. Consistent with the report by Jin et al. (2026) [33], all the bigel systems retained their ability to regain their original structure after the application of external forces.

### 3.5. Characterization of Bigel Based Foamed Emulsion

#### Foamability and Foam Stability

The overrun values and thermal stability values of the BFEs are given in Table 4. The influence of the gelatin concentration and SRP ratio on the overrun values were found to be statistically significant (*p* < 0.05). The 9% G-2 showed the highest overrun value while 6% G showed the lowest overrun value. Increases in the SRP ratio and gelatin concentration led to increased overrun. This could have been related to presence of saponin in the soapwort root powder and the presence of gelatin. Soapwort roots are one of the most well-known sources of triterpenoid saponins, with a total saponin concentration of 6.5% in the plant’s dry mass [57,58]. The substance known as saponin is what gives soapwort root powder its inherent surfactant qualities [59]. Saponin is also known to produce metastable foams with high foam stability [26,59]. Ozcan et al. (2022) [55] indicated that an increase in volume was found with a higher ratio of soapwort extract, indicating an enhancement in the foaming properties of the marshmallow foam, which was produced by the whipping process. However, gelatine possesses outstanding foaming, emulsifying, and gel-forming qualities, which are critical for stabilizing the air–water interface during whipping and producing a self-supporting gel-based foam [60,61]. The SRP and gelatine formed the foam with air bubbles. The bigel matrix’s strong viscoelastic gel strength (*G*′/elastic network) mechanically surrounded these air bubbles, preventing their escape and coalescence. Similar to these results, lowering the gelatin content resulted in a substantial decrease in intermolecular hydrogen bonds, diminishing the mechanical strength of the foam’s “backbone” [60].

The 9% G-2 samples had higher overrun values compared to the other bigel samples (*p* < 0.05). The influence of the gelatin–SRP concentration might explain this finding on the overrun value of the samples.

Effective foam formation depends on the rheological properties of the gel matrix, particularly its ability to incorporate air; gels with very low or excessively high storage modulus have poor foaming capabilities [18,62]. For example, stronger biopolymer networks can eventually reduce volume expansion by limiting bubble formation and expansion [17,32]. Similarly, in this study, the *G*′ values of the 6% G and 9% G bigel samples were greater than those of the other samples; however, these bigel samples showed the lowest overrun values.

The main influence of the gelatin concentration and the main effect of SRP ratio on the thermal stability values were found to be statistically significant (*p* < 0.05). An increase in SRP ratio led to an increase in thermal stability, except for 9% G-2. As previously documented in the literature, saponin-stabilized emulsions have great stability due to the creation of interface layers with strong expansion elasticity, which inhibit droplet deformation and coalescence [63,64]. As previously reported in the literature, saponin-based systems have excellent thermal stability due to significant steric and electrostatic repulsion between droplets [63]. Previous researchers [17] stated that protein-containing BFEs exhibit dominant elasticity (*G*′ > *G*″) and greater elastic modulus, indicating a more cohesive network that can withstand high temperatures. However, foam stability was much enhanced in the combined G-SRP system because the two materials created a robust viscoelastic network that stabilized the foam lamellae. This increase in thermal stability is presumed to have stemmed from saponin in the SRP and the robust viscoelastic structure of gelatin. On the other hand, interactions can also have detrimental effects on 9% G-2. Similar results were reported by [65] for QS/β-LG mixtures.

The 9% G-1 and 6% G-2 showed the highest thermal stability values, while 6% G and 9% G showed the lowest thermal stability values. The 9% G-1 and 6% G-2 samples had higher thermal stability values compared to the other bigel-based foamed emulsion samples (*p* < 0.05). The influence of the gelatin- SRP concentration might explain this finding on the thermal stability value of the samples (*p* < 0.05). However, all BFEs showed 75% or higher thermal stability.

**Figure 5:**
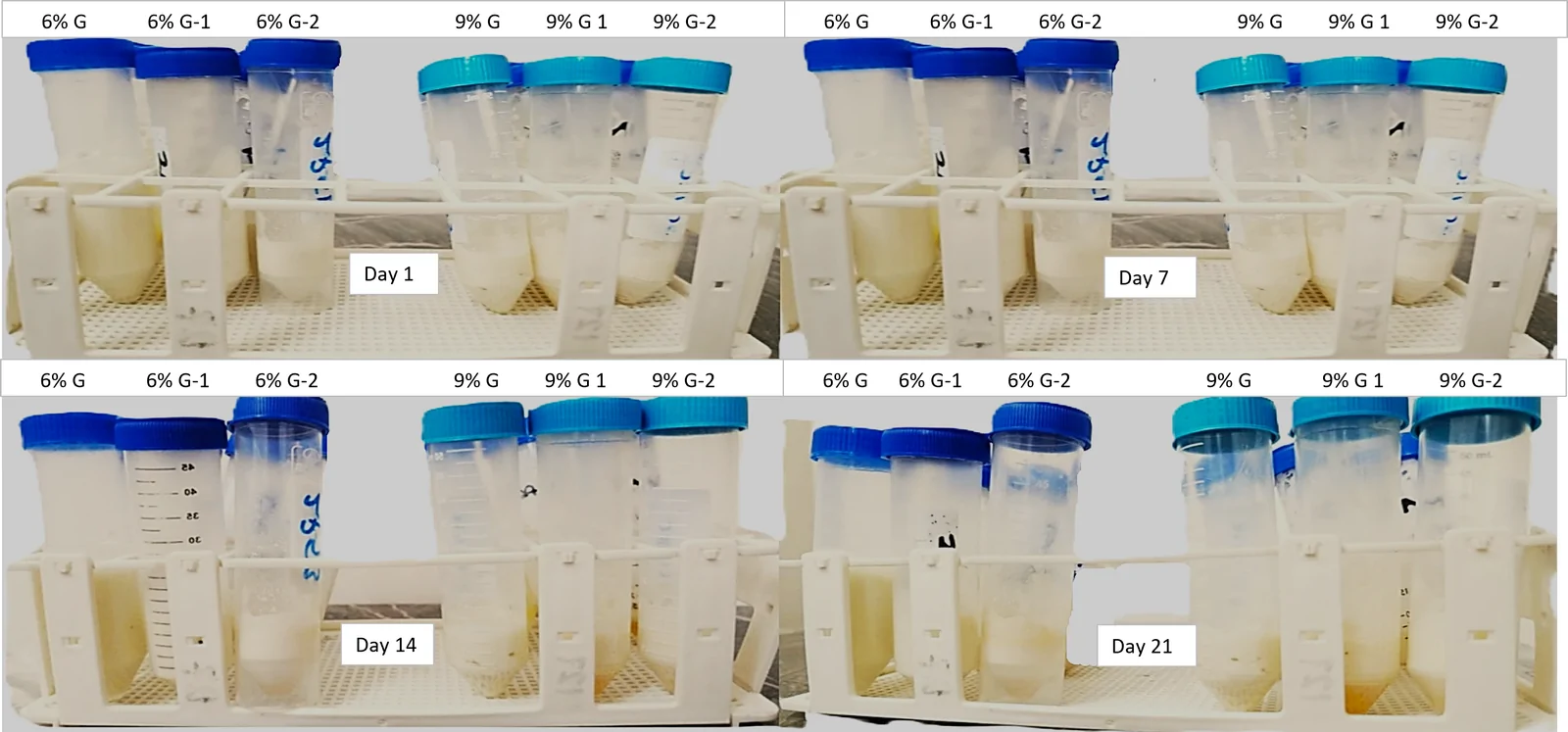
The Storage Stability of the Bigel-Based Foamed Emulsions (Samples were stored at approximately 20 °C and photographs were taken on Day 1, 7, 14, and 21).

To evaluate the stability over time of BFEs, samples were maintained at room temperature for 21 days following whipping. Figure 5 demonstrates that on the first day of storage, all BFEs retained their initial capacity and showed no signs of structural damage or oil leaking. This result was aligned with the result reported by Salahi and Mohebbi (2025) [17] for whey protein isolate–monoglyceride bigel-based foamed emulsions.

When the emulsions were visually examined throughout storage, emulsions with SRP over the period until day 7 showed no phase separation. It was observed that with prolonged storage of SRP, 9G1 and 9G2 precipitated to the bottom alongside the oil, starting from day 7, whereas 6G1 settled to the bottom from day 14 onwards. Although overrun was higher in 9% G-1 and 9% G-2, storage stability was lower as observed visually, compared to 6% G-1 and 6% G-2. Theoretically, this might be due to Laplace pressure; specifically, larger bubbles may have poorer sphericity and be more readily deformed than smaller ones due to the Laplace pressure inside the bubble droplets and growing bubble diameter, as previously reported in the literature [66].

### 3.6. Rheological Properties of the Mousse Samples

**Figure 6:**
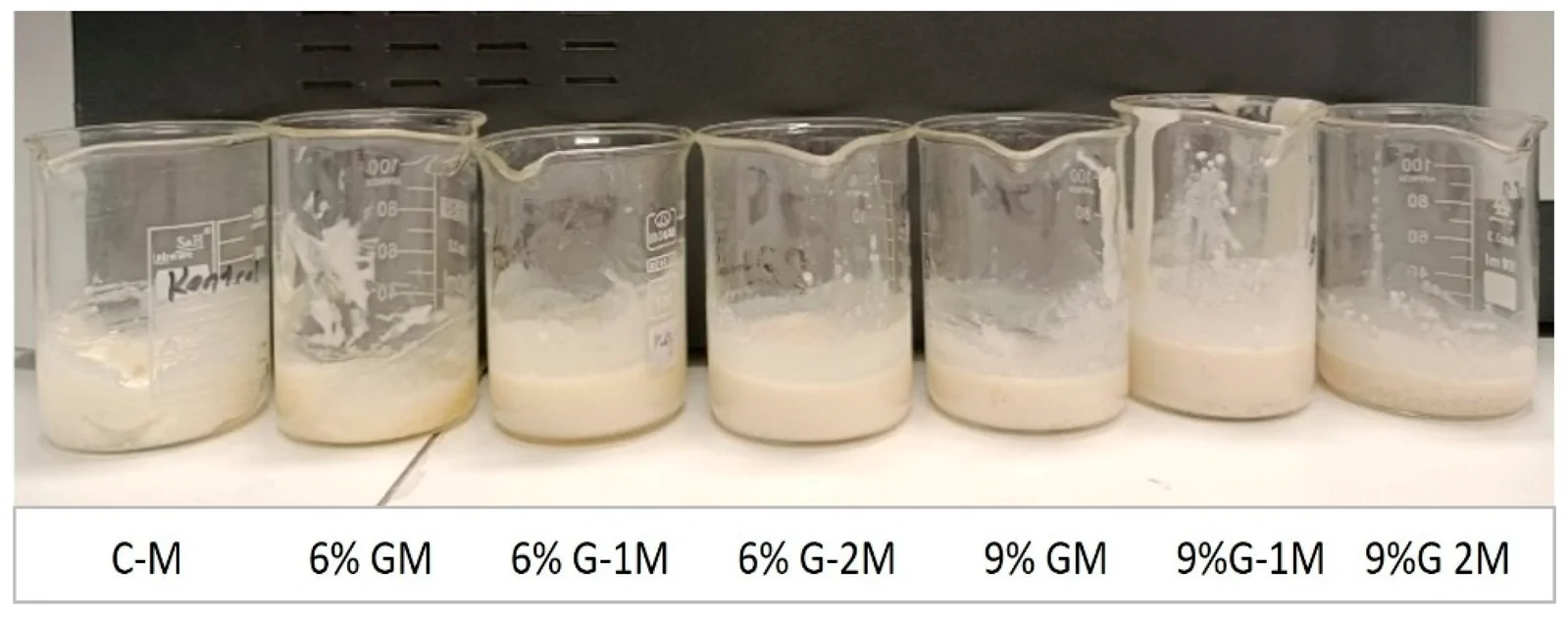
Visual Appearance of Mousse Samples.

**Figure 7:**
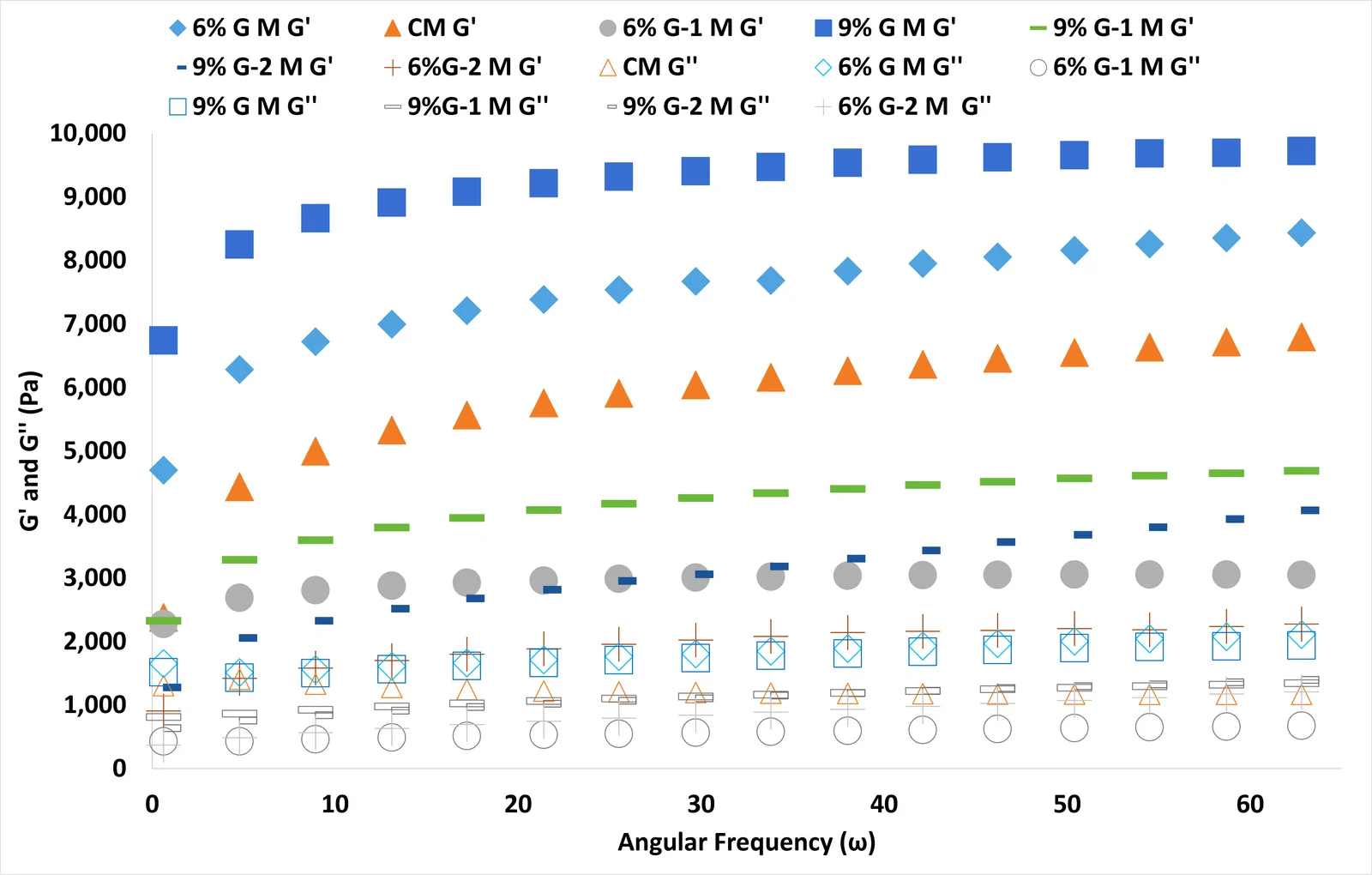
The Viscoelastic Properties of the Mousse Sample (mean, n = 3).

Figure 6 depicts the mousse samples’ appearance. Following inversion, none of the samples displayed fluidity. As seen in Figure 7, *G*′ values were greater than *G*″ values, and the mousse samples displayed solid-like behavior.

The 9% G M showed the highest *G*′ value and the 6% G-2 M showed the lowest *G*′ value. The *G*′ value increased as the concentration of gelatin increased and increase in SRP ratio led to decrease in *G*′ value. The bigel samples showed higher *G*′, *G*″, *K*′ and *K*″ values than the mousse samples. This might have been due to the combination of two-step mechanical disruption (foaming and folding) and phase dilution. This phenomenon may be explained by the higher shear rates observable in the colloidal gel network during mechanical foaming, which disrupted the attractive interactions among particles and led to individual particles adhering to the liquid–air interface during air absorption, as previously reported in the literature [66,67]. Once foaming ceased, the excess of surfactant promptly reestablished itself in a stationary condition, encasing each absorbed air bubble, which aligns with the literature [17]. As documented in the literature, adsorbed protein molecules prevent surfactant molecules from moving laterally in protein–surfactant systems, and surfactant molecules decrease the viscoelastic network of proteins [64]. This could help explain why the increase in SRP concentration led to decrease in *G*′ values of the mousse matrix. However, the *G*′ value increased as the concentration of gelatin increased. This can be explained by hydrogen bonding and van der Waals forces. Gelatin, a hydrophilic protein that forms robust networks, established a dense three-dimensional structure with the crystalline frameworks of beeswax in the oleogel [6,51].

The Power Law model was used to compare the findings regarding the dynamic rheological characteristics of the mousse samples (Table 5). *K*′ values were found to be higher than *K*″ values in every sample, confirming viscoelastic solid behavior. This model correctly simulated the dynamic rheological characteristics of the *K*″ values of the mousse samples except CM, 6% G M and 9% G M, as seen in Table 5. The main influence of the gelatin concentration and the main effect of SRP ratio on the *K*′ values were found to be statistically significant (*p* < 0.05). The *K*′ values of mousse samples ranged from 1046.3 to 7263.17 Pa·s. The *K*′ value of 9% G M was higher than that of the other BFE-based mousse samples. Additionally, a decrease in *K*′ value (*p* < 0.05) was caused by an increase in SRP concentration. On the other hand, the *K*′ value increased as the concentration of gelatin increased.

*K*″ values were greater in 6% G M and 9% G M than in the other samples. *K*″ values increased as the SRP ratio increased, with the exception of the 6% and 9% G-2 M samples. Unlike the bigel results, the gelatin concentration influenced the *K*″ values of the mousse samples *p* < 0.05.

The 6% G-1 M and 9% G-1 M were determined to be the closest to the control mousse sample in terms of *K*′ value, when comparing BFE mousse samples with the control mousse sample (*p* > 0.05). However, 6% G M and 9% G M were determined to be the closest to the control mousse sample in terms of *K*″ value when comparing BFE mousse samples with the control mousse sample (*p* > 0.05).

### 3.7. Textural Properties of the Mousse Samples

Table 6 displays the mousses’ textural characteristics, including gumminess, chewiness, springiness, cohesiveness, and hardness. The chewiness values of mousse samples increased with gelatin concentration and SRP ratio (*p* < 0.05). Gumminess and hardness were improved by increasing the mousse’s gelatin concentration and SRP ratio from 1 to 2 (*p* < 0.05). The 6% G-2 M and 9% G-2 M samples showed the highest hardness and chewiness values (*p* < 0.05). Also, the 9% G-2 M sample showed the highest gumminess value (*p* < 0.05). The hardness value rose in accordance with the SRP concentration. Mousse with 9% G-2 M was the hardest sample, while 6% G M was the softest. The increased gel strength and viscoelastic network could be attributed to the incorporation of SRP and gelatin potential function as active fillers through hydrogen bonding and/or hydrophobic interactions [51]. The bigel’s discrete phase functioned as an “active filler,” enhancing hardness and enabling better gel structure [52,53], which could explain the higher hardness values within the mousse. The cohesiveness values of the BFEs were found to be between 0.520 and 0.732. The increased mechanical performance resulting from the integration of SRP and gelatin is likely to have been due its network-forming characteristics, which generated a resilient gel structure that efficiently filled cavities and increased the overall mechanical stability of the system [5]. Similar findings in terms of hardness and cohesiveness were recorded by Salahi and Mohebbi (2026) [5] for low-acyl gellan gum bigel.

An increase in SRP ratio resulted in an increase in hardness, gumminess, and chewiness values, except for the 9% G-1 M sample. Previous research [68] reported that when diacylglycerol-based solid lipid nanoparticles’ concentration was 2.5 wt %, the particles were insufficient to cover the interface and form network support. As previously reported in the literature, the insufficient interfacial adsorption of surfactant molecules at the gas–liquid interface may have led to the gas–liquid interface becoming more fragile [69], which could help explain the observed result.

The elasticity that defines the rubbery sensation of bigel-based foams in the mouth signifies their elastic recovery ability. The 6% G-2 M showed the highest springiness value and no statistically significant difference from the CM. All mousse samples showed high springiness values (0.921–0.987), indicating that the mousse samples possessed high elasticity. Previous research [60] indicated that all bigel-based foams exhibit high elasticity, with an elasticity value of around 1.

The 6% G-2 M was determined to be the closest to the control mousse sample in terms of hardness, springiness, cohesiveness, and gumminess values when the BFE mousse samples were compared with the control mousse sample (*p* > 0.05).

### 3.8. Color Properties of the Mousse Samples

Table 7 shows the color characteristics of the mousse samples. The mousse samples’ L* values varied between 76.78 and 88.38. The sample with the highest L* value (88.38) was the control mousse. The gelatin–SRP ratio had no statistically significant effect on the L* values of the mousse samples, with the exception of 6% G M and 9% G-2 M.

The samples’ b* values varied from 6.10 to 11.34. The highest (11.34) and lowest b* value were found in the 6% G-1 M and 9% G-1 M, respectively. A color that is closer to yellow is indicated by a greater b* value.

The samples’ a* values ranged from −0.68 to −2.32. All formulations had negative values, signifying a transition towards green tones. The most negative a* value (−2.32) was seen in the 6% G M samples. The least negative a* value (−0.68) was seen in the 9% G-2 M. An increase in gelatin concentration led to a less negative a* value in the samples (*p* < 0.05). An increase in SRP ratio from 1 to 2 led to a less negative a* value (*p* < 0.05). The influence of the main effect of gelatin concentration on the a* and b* values was found to be statistically significant. Overall, an increase in gelatin concentration from 6% to 9% led to an increase in a* value (which became less negative) and a decrease in the b* values of the mousse samples across all SRP concentrations.

No specific trend or regular change was observed in the L*, a*, or b* color parameters of the samples depending on the formulation. The samples’ ΔE* values varied from 5.95 to 11.93. The 6% G-2 M sample showed the lowest ΔE* value and was the closest sample to CM. The gelatin–SRP ratio had no significant effect on the ΔE* values of the mousse samples, with the exception of 6% G M and 9% G-2 M. The 6% G M and 9% G-2 M samples showed the highest ΔE* values (*p* < 0.05).

## 4. Conclusions

This study showed that the use of gelatine–SRP–beeswax for producing bigels and bigel-based foamed emulsions improved viscoelasticity, overrun, thermal stability, and the production of aerated structures. The 6% G-2 M sample showed the closest instrumental similarity to the control mousse in terms of its selected physicochemical properties. These results demonstrate that gelatin–SRP-enhanced BFEs provide an excellent strategy to reduce fat content while preserving the required functional characteristics for use in mousse fabrication. In summary, bigel-based foamed emulsion can serve as an appropriate substitute for cream in mousse formulation. The main limitations of the study are the lack of nutritional composition, sensory evaluation, and final product storage assessment. Future studies could evaluate the sensory acceptability, nutritional composition, oxidative stability, and storage performance of the optimized BFE-based mousse.

## Figures and Tables

**Table 1:** Formulations of Bigel Samples.

Sample	Avocado Oil Beeswax Oleogel (%)	Gelatin Hydrogel (%)	SRP (%)	Hydrogel:Oleogel Ratio
6% G	8	6	0	50:50
6% G-1	8	6	1	50:50
6% G-2	8	6	2	50:50
9% G	8	9	0	50:50
9% G-1	8	9	1	50:50
9% G-2	8	9	2	50:50

6% G: 6% gelatin hydrogel bigel; 6% G-1: 6% gelatin hydrogel with 1% soapwort root powder bigel, 6% G-2: 6% gelatin hydrogel with 2% soapwort root powder bigel, 9% G: 9% gelatin hydrogel bigel, 9% G-1: 9% gelatin hydrogel with 1% soapwort root powder bigel, 9% G-2: 9% gelatin hydrogel with 2% soapwort root powder bigel.

**Table 2:** Dynamic Rheological Properties of Bigels.

Sample	*K*′ (Pa·s^n^)	*n*′	*R^2^*	*K*″ (Pa·s^n^)	*n*″	*R^2^*
6% G	10,324.20 ± 487.59 BCay	0.036 ± 0.008	0.721 ± 0.084	2124.14 ± 133.59 Aax	0.123 ± 0.008	0.912 ± 0.064
6% G-1	8312.95 ± 903.24 Cay	0.054 ± 0.005	0.952 ± 0.008	1041.75 ± 93.78 Cbx	0.147 ± 0.003	0.897 ± 0.011
6% G-2	8346.74 ± 131.53 Cay	0.080 ± 0.021	0.944 ± 0.008	1111.47 ± 103.93 Cbx	0.229 ± 0017	0.923 ± 0.014
9% G	13,267.87 ± 16.87 ABax	0.065 ± 0.021	0.980 ± 0.014	1630.30 ± 1.49 Bax	0.144 ± 0.008	0.949 ± 0.002
9% G-1	14,019.41 ± 358.89 Aax	0.040 ± 0.000	0.930 ± 0.042	1415.68 ± 88.94 BCbx	0.112 ± 0.028	0.783 ± 0.028
9% G-2	12,364.07 ± 1818.76 ABax	0.050 ± 0.014	0.937 ± 0.008	1070.20 ± 160.03 Cbx	0.193 ± 0.018	0.906 ± 0.007

Two-Way ANOVA and Tukey’s HSD test were used to assess statistical differences (*p* < 0.05). Significant differences are indicated by different uppercase letters (A, B) for the interaction effect of gelatin x soapwort root powder concentration, different lowercase letters (a, b) for the main effect of goapwort root powder concentration, and different lowercase letters (x, y) for the main effect of gelatin concentration. 6% G: 6% gelatin hydrogel bigel, 6% G-1: 6% gelatin hydrogel with 1% soapwort root powder bigel, 6% G-2: 6% gelatin hydrogel with 2% soapwort root powder bigel, 9% G: 9% gelatin hydrogel bigel, 9% G-1: 9% gelatin hydrogel with 1% soapwort root powder bigel, 9% G-2: 9% gelatin hydrogel with 2% soapwort root powder bigel.

**Table 3:** Textural Properties of Bigel Samples.

	Hardness (N)	Springiness	Cohesiveness	Gumminess	Chewiness
6% G	0.204 ± 0.004 Ecy	0.995 ± 0.001 Aax	0.550 ± 0.026 ABay	0.112 ± 0.004 Dcy	0.111 ± 0.004 Ecy
6% G-1	0.675 ± 0.023 Dby	0.992 ± 0.011 Abx	0.484 ± 0.015 Bay	0.326 ± 0.009 Cby	0.323 ± 0.010 Dby
6% G-2	0.835 ± 0.020 Cay	0.992 ± 0.009 Aax	0.476 ± 0.004 Bay	0.398 ± 0.007 BCay	0.395 ± 0.007 CDay
9% G	0.925 ± 0.026 Ccx	0.998 ± 0.001 Aay	0.520 ± 0.003 ABax	0.481 ± 0.013 Bcx	0.480 ± 0.013 BCcx
9% G-1	1.171 ± 0.052 Bbx	0.858 ± 0.099 Bby	0.569 ± 0.058 Aax	0.667 ± 0.084 Abx	0.579 ± 0.137 Bbx
9% G-2	1.378 ± 0.061 Aax	0.997 ± 0.001 Aay	0.538 ± 0.007 ABax	0.742 ± 0.031Aax	0.740 ± 0.031 Aax

Two-Way ANOVA and Tukey’s HSD test were used to assess statistical differences (*p* < 0.05). Significant differences are indicated by different uppercase letters (A, B) for the interaction effect of gelatin x soapwort root powder concentration, different lowercase letters (a, b) for the main effect of soapwort root powder concentration and different lowercase letters (x, y) for the main effect of gelatin concentration. 6% G: 6% gelatin hydrogel bigel, 6% G-1: 6% gelatin hydrogel with 1% soapwort root powder bigel, 6% G-2: 6% gelatin hydrogel with 2% soapwort root powder bigel, 9% G: 9% gelatin hydrogel bigel, 9% G-1: 9% gelatin hydrogel with 1% soapwort root powder bigel, 9% G-2: 9% gelatin hydrogel with 2% soapwort root powder bigel.

**Table 4:** The Foam Stability and Thermal Stability (%) of the Bigel-Based Foamed Emulsions.

	Overrun (%)	Thermal Stability (%)
6% G	2.40 ± 0.17 Ecy	75.39 ± 0.19 Dcy
6% G-1	21.03 ± 0.10 Dby	89.31 ± 0.08 Cby
6% G-2	24.55 ± 0.26 Cay	98.62 ± 0.14 Aay
9% G	24.54 ± 0.10 Ccx	75.42 ± 0.21 Dcx
9% G-1	36.86 ± 0.24 Bbx	98.31 ± 0.21 Abx
9% G-2	48.32 ± 0.38 Aax	94.26 ± 0.80 Bax

Two-Way ANOVA and Tukey’s HSD test were used to assess statistical differences (*p* < 0.05). Significant differences are indicated by different uppercase letters (A, B) for the interaction effect of gelatin x soapwort root powder concentration, different lowercase letters (a, b) for the main effect of soapwort root powder concentration, and different lowercase letters (x, y) for the main effect of gelatin concentration. 6% G: 6% gelatin hydrogel bigel based foamed emulsion, 6% G-1: 6% gelatin hydrogel with 1% soapwort root powder bigel based foamed emulsion, 6% G-2: 6% gelatin hydrogel with 2% soapwort root powder bigel based foamed emulsion, 9% G: 9% gelatin hydrogel bigel based foamed emulsion, 9% G-1: 9% gelatin hydrogel with 1% soapwort root powder bigel based foamed emulsion, 9% G-2: 9% gelatin hydrogel with 2% soapwort root powder bigel based foamed emulsion.

**Table 5:** Dynamic Rheological Properties of Mousse Samples.

Sample	*K*′ (Pa·s^n^)	*n*′	*R^2^*	*K*″ (Pa·s^n^)	*n*″	*R^2^*
CM	3179.65 ± 242.58 -	0.188 ± 0.021	0.985 ± 0.001	1370.34 ± 60.93 -	0.036 ± 0.002	0.79 ± 0.026
6% G M	5170.66 ± 346.94 Bay *	0.119 ± 0.020	0.993 ± 0.007	1421.31 ± 107.64 Aay -	0.079 ± 0.012	0.783 ± 0.050
6% G-1 M	2429.22 ± 514.24 Cby -	0.062 ± 0.026	0.914 ± 0.066	371.32 ± 83.17 Bby **	0.134 ± 0.024	0.924 ± 0.019
6% G-2 M	1046.30 ± 194.24 Ccy **	0.194 ± 0.051	0.993 ± 0.005	267.32 ± 46.04 Bby **	0.353 ± 0.051	0.979 ± 0.004
9% G M	7263.17 ± 1634.25 Aax *	0.074 ± 0.005	0.988 ± 0.000	1355.15 ± 362.49 Aax -	0.080 ± 0.014	0.868 ± 0.036
9% G-1 M	2592.82 ± 493.59 Cbx -	0.144 ± 0.011	0.998 ± 0.002	721.62 ± 177.31 Bbx **	0.139 ± 0.006	0.951 ± 0.004
9% G-2 M	1278.28 ± 222.78 Ccx **	0.268 ± 0.004	0.991 ± 0.001	550.58 ± 84.65 Bbx **	0.215 ± 0.001	0.977 ± 0.002

Two-Way ANOVA and Tukey’s HSD test were used to assess statistical differences (*p* < 0.05). Significant differences are indicated by different uppercase letters (A, B) for the interaction effect of gelatin x soapwort root powder concentration, different lowercase letters (a, b) for the main effect of soapwort root powder concentration and different lowercase letters (x, y) for the main effect of gelatin concentration. Dunnett’s test was used to statistically compare the experimental formulations with the control group (CM); (*) denotes values significantly higher than the control, (**) indicates values significantly lower than the control (*p* < 0.05), and (-) indicates no statistically significant difference compared to the control group. CM: mousse formulation prepared with whipped cream, 6% G M: 6% gelatin hydrogel gigel-based foamed emulsion mousse, 6% G-1 M: 6% gelatin hydrogel with 1% soapwort root powder bigel-based foamed emulsion mousse, 6% G-2 M: 6% gelatin hydrogel with 2% soapwort root powder bigel-based foamed emulsion mousse, 9% G M: 9% gelatin hydrogel bigel-based foamed emulsion mousse, 9% G-1 M: 9% gelatin hydrogel with 1% soapwort root powder bigel-based foamed emulsion mousse, 9% G-2 M: 9% gelatin hydrogel with 2% soapwort root powder bigel-based foamed emulsion mousse.

**Table 6:** Textural Properties of Mousse Samples.

Mousse Texture Profile					
	Hardness (N)	Springiness	Cohesiveness	Gumminess	Chewiness
CM	0.812 ± 0.164 -	0.968 ± 0.006 -	0.548 ± 0.002 -	0.446 ± 0.092 -	0.432 ± 0.091 -
6% G M	0.109 ± 0.009 Dby **	0.972 ± 0.008 Aabx -	0.632 ± 0.026 ABbx -	0.069 ± 0.008 Eby **	0.067 ± 0.007 Dcy **
6% G-1 M	0.378 ± 0.007 Cby **	0.921 ± 0.003 Bbx **	0.592 ± 0.020 ABbx -	0.224 ± 0.009 Dby **	0.206 ± 0.008 Cby **
6% G-2 M	0.768 ± 0.070 Abay -	0.987 ± 0.004 Aax -	0.662 ± 0.015 ABax -	0.509 ± 0.053 Bay -	0.545 ± 0.047 Aay *
9% G M	0.638 ± 0.117 Bbx -	0.951 ± 0.037 ABabx -	0.520 ± 0.012 Bbx -	0.331 ± 0.053 Cbx **	0.313 ± 0.039 Bcx **
9% G-1 M	0.422 ± 0.093 Cbx **	0.984 ± 0.005 Abx -	0.583 ± 0.108 Bbx -	0.262 ± 0.036 CDbx **	0.257 ± 0.036 Cbx **
9% G-2 M	0.857 ± 0.128 Aax -	0.973 ± 0.005 Aax -	0.732 ± 0.082 Aax *	0.620 ± 0.027 Aax *	0.604 ± 0.023 Aax *

Two-Way ANOVA and Tukey’s HSD test were used to assess statistical differences (*p* < 0.05). Significant differences are indicated by different uppercase letters (A, B) for the interaction effect of gelatin x soapwort root powder concentration, different lowercase letters (a, b) for the main effect of soapwort root powder concentration and different lowercase letters (x, y) for the main effect of gelatin concentration. Dunnett’s test was used to statistically compare the experimental formulations with the control group (CM); (*) denotes values significantly higher than the control. (**) indicates values significantly lower than the control (*p* < 0.05). and (-) indicates no statistically significant difference compared to the control group. CM: mousse formulation prepared with whipped cream, 6% G M: 6% gelatin hydrogel gigel-based foamed emulsion mousse, 6% G-1 M: 6% gelatin hydrogel with 1% soapwort root powder bigel-based foamed emulsion mousse, 6% G-2 M: 6% gelatin hydrogel with 2% soapwort root powder bigel-based foamed emulsion mousse, 9% G M: 9% gelatin hydrogel bigel-based foamed emulsion mousse, 9% G-1 M: 9% gelatin hydrogel with 1% soapwort root powder bigel-based foamed emulsion mousse, 9% G-2 M: 9% gelatin hydrogel with 2% soapwort root powder bigel-based foamed emulsion mousse.

**Table 7:** The Color Properties of the Mousse Samples.

	L*	a*	b*	ΔE*
CM	88.38 ± 0.51	−1.63 ± 0.07	6.10 ± 0.51	-
6% G M	77.44 ± 0.69 Bax **	−2.32 ± 0.10 Cby *	9.78 ± 0.79 ABCax *	11.59 ± 0.42 Aax
6% G-1 M	82.01 ± 0.87 Aax **	−2.10 ± 0.12 Cby *	11.34 ± 1.08 Aax *	8.27 ± 1.33 Bax
6% G-2 M	84.37 ± 1.12 Aax **	−0.81 ± 0.03 ABay **	10.37 ± 0.14 ABax *	5.95 ± 0.80 Bax
9% G M	83.15 ± 1.13 Aax **	−0.83 ± 0.06 ABbx **	9.95 ± 0.21 ABCay *	6.58 ± 0.82 Bax
9% G-1 M	81.41 ± 1.91 Aax **	−1.07 ± 0.13 Bbx **	8.10 ± 1.02 Cay *	7.37 ± 1.61 Bax
9% G-2 M	76.78 ± 1.57 Bax **	−0.68 ± 0.15 Aax **	8.57 ± 0.75 BCay *	11.93 ± 1.41 Aax

Two-Way ANOVA and Tukey’s HSD test were used to assess statistical differences (*p* < 0.05). Significant differences are indicated by different uppercase letters (A, B) for the interaction effect of gelatin x soapwort root powder concentration, different lowercase letters (a, b) for the main effect of soapwort root powder concentration and different lowercase letters (x, y) for the main effect of gelatin concentration. Dunnett’s test was used to statistically compare the experimental formulations with the control group (CM); (*) denotes values significantly higher than the control. (**) indicates values significantly lower than the control (*p* < 0.05). and (-) indicates no statistically significant difference compared to the control group. CM: mousse formulation prepared with whipped cream, 6% G M: 6% gelatin hydrogel gigel-based foamed emulsion mousse, 6% G-1 M: 6% gelatin hydrogel with 1% soapwort root powder bigel-based foamed emulsion mousse, 6% G-2 M: 6% gelatin hydrogel with 2% soapwort root powder bigel-based foamed emulsion mousse, 9% G M: 9% gelatin hydrogel bigel-based foamed emulsion mousse, 9% G-1 M: 9% gelatin hydrogel with 1% soapwort root powder bigel-based foamed emulsion mousse, 9% G-2 M: 9% gelatin hydrogel with 2% soapwort root powder bigel-based foamed emulsion mousse.

## Data Availability

The data that support the findings of this study are available from the corresponding author upon reasonable request.
